# Comparison of Post-Operative Muscular Strength Between Gamma Nailing and Hemiarthroplasty System in Femoral Intertrochanteric Fractures

**DOI:** 10.2174/1874325001711010255

**Published:** 2017-03-31

**Authors:** Mitsuaki Noda, Yasuhiro Saegusa, Masayasu Takahashi, Chisa Noguchi, Chihiro Yoshikawa, Hiroshi Mikami, Akira Gotouda

**Affiliations:** 1 Konan Hospital, Department of Orthopedics, Kobe, Japan; 2 Yoshinogawa Medical Center, Department of Rehabilitation, Yoshinogawa city, Japan

**Keywords:** Bipolar hip prosthesis, Cephalo medullary nailing, Comparative study, Femoral intertrochanteric fracture, Gluteus medius, Muscle strength

## Abstract

**Background:**

The current study focuses on the comparison of postoperative muscular strength around the hip joint of patients with femoral intertrochanteric fractures treated either by cephalo-medullary (CM) nailing or a new bipolar hip prosthesis (BHP), an especially attached device to secure displaced greater trochanteric fragment.

**Methods:**

Twenty patients treated with CM nailing were age- and sex- matched with a control group of 20 patients treated with BHP. Maximum isometric forces at the bilateral hip joint were measured during the follow up period. Means of 3 measurements were represented.

**Results:**

The mean and standard deviation values (kg) of muscle strength at the non-operative/ operative side in the CM nailing group were as follows: flexion strength 9.5±4.7/8.5±4.9 (P=0.06), extension strength 6.2±3.5/5.5±3.7 (P=0.08), abduction strength at 0 degrees 7.7±3.5/6.2±2.8 (p=0.002), abduction strength at 10 degrees 5.5±2.0/4.2±2.0 (p=0.001). In the BHP group, mean and standard deviation values of muscle strength at the non-operative/ operative side were as follows: flexion strength 6.5±2.8/6.0±3.4 (P=0.08), extension strength 4.4±0.9/4.4±0.6 (P=0.83), abduction strength at 0 degrees 5.1±1.9/5.0±1.6 (p=0.12), and that at 10 degrees 4.7±1.4/4.6±1.3 (p=0.10).

**Conclusion:**

Our results demonstrate that CM nailing may cause a 25-30% decrease in postoperative muscle strength around the hip joint, particularly during hip abduction. With the new BHP, greater trochanter reduction is achieved allowing early weight bearing and maintaining strength in abduction. Surgeons should consider postoperative muscular strength as one of the necessary factors for selection of the appropriate surgical procedure.

**Level of Evidence:**

Therapeutic Level III.

## INTRODUCTION

Cephalo-medullary (CM) nailing is the surgical gold standard treatment for femoral intertrochanteric fractures [1, 2]. From a biomechanical standpoint, an intramedullary device with a shorter lever arm can favorably transfer weight bearing force into controlled sliding. Sliding towards fracture fragments will promote fracture healing and stabilize the fragments, thus enabling early rehabilitation and weight-bearing on the fractured extremity [3]. However, shortening via sliding may cause decreased muscle strength, especially of the gluteus medius, based on muscular physiology [4]. In our outpatient clinic experience, we presume that decreased muscle strength, especially of the gluteus medius, is one of the causes of postoperative functional impairment. This opinion correlates with a report stating that only 55% of patients regained pre-fracture walking ability, and 65% returned to the pre-fracture ADL level at the two-year follow-up period [5].

The use of bipolar hip prosthesis (BHP) has been advocated by some surgeons, especially for unstable intertrochanteric fractures [6]. This procedure does not cause excessive femoral shortening allowing retention of gluteus muscular strength [7, 8], which is favorable for immediate postoperative weight-bearing. Although, occurrence of greater trochanter non-union using conventional plates or wires in BHP was allegedly high [9], a new type of prosthesis with an attachment to stabilize the displaced greater trochanter has been designed by one of our coauthors, expecting sufficient purchase of greater trochanter and maintenance of muscle strength of the gluteus medius. So far, several comparative studies regarding operative invasiveness or postoperative complications between implant types for intertrochanteric fractures have been done [10, 11]. However, to our knowledge none of the articles presented results measuring postoperative muscular strength.

The current study focuses on postoperative muscular strength of the hip joint after CM nailing or bipolar hip prosthesis, comparing the non-operative to the operative side, without reference to intra- and post-operative complications. We hypothesized that BHP treatment could provide a significant positive effect on retaining postoperative muscle strength, especially abduction force.

## MATERIALS AND METHODS

A total of 80 patients with femoral intertrochanteric fractures treated with cephalo-medullary femoral nail implants from 2012 March to 2014 September were extracted from the trauma database of Konan Hospital. Exclusion criteria included: (1) less than 70 years old at time of surgery, (2) less than eleven months total follow-up period, (3) bilaterally operated hip, (4) dementia, (5) inability to walk/non-ambulatory, (6) central or spinal nerve disorder, or Parkinson’s disease as screened by the attending physician, (7) deceased, (8) more than five years postoperatively, and (9) any other pathology that may affect hip muscle power through physical examination and radiographic assessment.

After applying the exclusion criteria, 22 patients were left and 20 out of the 22 could joined in the study. Mean age at osteosynthesis with CM nailing was 84 years (range, 73 to 91 years), and the mean post-operative follow-up period was 580 days (range, 330 to 829 days). Two patients were male (10%). All patients had no major intra- and post-operative complications and completed the post-operative rehabilitation program.

A control group of 20 patients treated with BHP was selected from the Yoshinogawa Medical Center database to match the number of patients, sex distribution, and average age of surgery with the CM nailing group. Mean age at osteosynthesis was 86 years (range, 77 to 93 years), and the mean post-operative follow-up period was 555 days (range, 326 to 1514 days). Two patients were male (10%).

The intertrochanteric fracture type was classified as A-1 type (two part fractures) or A-2 type (multi-part fractures) based on the AO/OTA classification. In the CM nailing group, 10 patients had A-1 and another 10 patients had A-2 fractures. In the control BHP group, 8 patients were A-1 type and twelve were A-2 type. Patient demographics are summarized in Table **1**.

## OPERATIVE TECHNIQUE FOR EACH IMPLANT

CM nailing was performed under conventional procedure, and all 20 patients received the same type of short nail implant (Targon PF nail ^®^, B. B. Aesculap, Tuttlingen, Germany), consisting of two screws into the femoral head. During surgery, a 16.5mm diameter hole created over the tip of greater trochanter might invite considerable damage on gluteus medius.

**Fig. (1):**
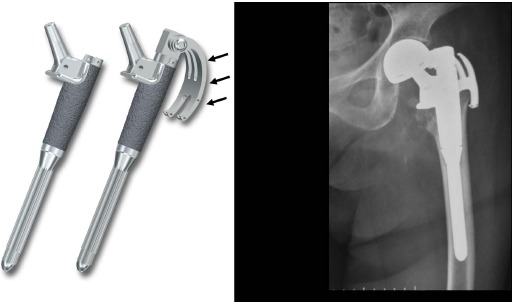
A novel bipolar hip prosthesis (BHP) implant characterized by an attachment to control upper translation forces of the greater trochanter (arrows) and its radiogram (right).

For the BHP procedure, all 20 patients received a modular hip stem implant (MOD-centaur ^®^, Kyocera medical Co, Osaka, Japan) (Fig. **1**). Preoperative planning was crucial to scale the appropriate length of the stem by comparing the center of the injured hip with the contralateral non-injured side. A posterolateral modified Gibson approach was performed. Proximal femoral cuts around the cervical area were fashioned by laying the cutting guide along the posterior aspect of the femur or by utilizing the trial prosthesis as a guide. After removing the femoral head, remnants of proximal fragments were cleaned out to accommodate the prosthesis. The trial prosthesis with calcar is available in three lengths (34, 45, and 55 mm) to maintain limb length and femoral offset. It is necessary to have a stable platform to position the prosthesis by leveling medial cortex. Anteversion-retroversion of the prosthesis was determined as a reference to the posterior aspects of the medial and lateral femoral condyles. An especially equipped hook to reduce the displaced greater trochanter fragment to its original position was attached to the prosthesis to retain physiological tension of the gluteus medius. Longitudinal spit at gluteus medius muscle was made to connect the hook to the prosthesis. This bony fragment was trimmed if necessary, so that the hook can well hold it. Upon confirmation that the trial was properly set during adequate passive range of motion, the prosthesis was implanted. No other additional wires or materials were necessary to support the displaced fragment.

Preoperative bed side rehabilitation was started on the day of hospitalization. Postoperative rehabilitation program of both groups was basically same: muscular coordination, strength training, and early weight bearing without limitation using walker or a cane for more than 40 minutes a day, as supervised by a physical therapist. In addition, patients were encouraged to walk on the hospital corridors with aid of nurses, once their condition becomes stable. The average period of postoperative hospitalization differed in each hospital: almost a few months in CM nailing group, and about three weeks in BHP group later transferred to other facilities. This difference was based on dissimilar hospital management. At outpatients’ clinic, patients are recommended to follow up period, as long as they can walk.

## MEASUREMENT OF MUSCULAR STRENGTH

Maximum isometric forces around the hip in flexion, extension, and abduction at angles of 0 and 10 degrees, as well as a routine physical examination were measured during the follow-up period. Muscle strength was recorded with a handheld dynamometer (μ tas F-1, Anima, Tokyo) attached on femoral supracondylar lesion, based on Katoh M *et al.* [12]. Two senior physical therapists (C.Y. and A.G.) directed all the measurement in the two hospitals to escape bias for measuring. Subjects were stabilized using a pelvic strap in supine or prone position, or sustained in the back in a sitting position. Muscular strength on hip flexion was measured with the patient in the sitting position. Extension strength was evaluated in prone position with the hip flexed at the contralateral side [13]. Abduction strength was examined in the supine position with the hip and knee extended. The legs were maintained in internal rotation to prevent activation of the quadriceps muscle that may compensate for hip abduction. Using a goniometer, patients’ contralateral leg was positioned so as not to add counteracting forces bringing erroneous data. In all measurements, they first performed sub-maximum contraction to accustom to the testing. Then, they were instructed to maintain maximum force for 5 seconds. Therapists always paid attention to them not to get tired, and slowly proceeded measurement with spending enough time to refresh. Means of 3 measurements per position were recorded, and values between the non-operated and operated sides were compared and analyzed.

## VERTICAL SHORTENING AT FRACTURE SITE

Shortening of the femoral intertrochanteric fracture in anterolateral view radiographs was defined as vertical translation of the femoral head against the femoral shaft. Values were calculated by a single author (MN) using the formula: [Parameter non-fracture side - Parameter fracture side], as a difference from the non-operated to the operated side with each implant. Even among patients with less shortening, values of muscular strength were compared for statistical significance.

## STATISTICAL ANALYSES

All data were analyzed using the Statistical Package for the Social Sciences software program (SPSS, version 14.0, Tokyo, Japan). Mean and standard deviation values for each muscle strength were calculated from the non- operative and operative sides. Comparison of muscle strength between non-operative and operative sides was done with the Wilcoxon Signed Rank test.

## SOURCE OF FINDING

No external funding was received for this study.

## RESULTS

**Fig. (2):**
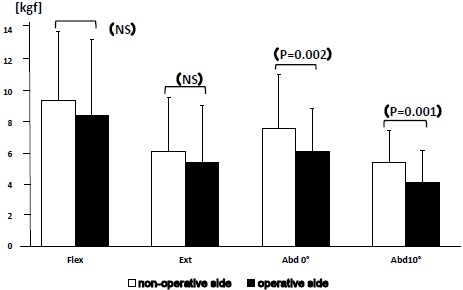
Bar graph depicting difference of mean muscle strength between the non-operated and operated side of the CM nailing group, in kilogram force (kgf). Only muscle strength during abduction showed statistically difference (P<0.01). Error bars indicate standard deviation.

Values of muscular strength on the operated side were generally lower than those in the non-operated side (Fig. **2**). Mean and standard deviation values (kgf) for muscle strength from the non-operative/ operative side in the CM nailing group are as follows: flexion strength 9.5±4.7/8.5±4.9 (P=0.06), extension strength 6.2±3.5/5.5±3.7 (P=0.08), abduction strength at 0 degrees 7.7±3.5/6.2±2.8 (p=0.002), abduction strength at 10 degrees 5.5±2.0/4.2±2.0 (p=0.001). Abduction strength at both 0 and 10 degrees were statistically lower in the operative side. Muscle strength values in abduction at 0 and 10 degrees were statistically lower in the operated side compared to the non-operated side.

**Fig. (3):**
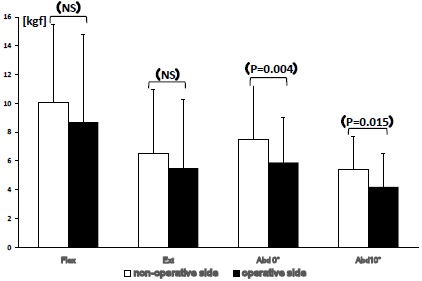
Bar graph showing values of abduction muscle strength between the non-operated and operated side in CM nailing patients with femoral neck shortening of 7 mm or less. Decrease in muscle strength values in the operated side during abduction at 0 and 10 degrees were statistically significant even in less displaced group (p<0.05).

The average length of vertical shortening of the fracture site in the CM nailing group was 5.9±5.3mm (0 to 20mm) based upon the contralateral side. A negative correlation between muscle strength and femoral neck shortening in the radiographs was not evident. Thirteen patients with shortening of 7mm or less were selected for further analysis. Hip muscle strength at the non-operated and the operated side were as follows: flexion 10.1±5.4/8.7±6.1 (P=0.05), extension 6.5±4.5/5.5±4.8 (P=0.08), abduction at 0 degrees 7.5±3.9/5.9±3.2 (p=0.004), abduction at 10 degrees 5.4±2.3/4.2±2.4 (p=0.015) (Fig. **3**).

**Fig. (4):**
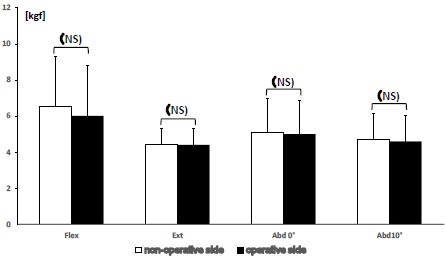
Bar graph representing the difference in mean muscle strength between the non-operated and operated sides of BHP patients. No statistical difference was founded.

In the BHP group, mean and standard deviation values for muscle strength from the non-operative/operative side are as follows: flexion strength 6.5±2.8/6.0±3.4 (P=0.08), extension strength 4.4±0.9/4.4±0.6 (P=0.83), abduction strength at 0 degrees 5.1±1.9/5.0±1.6 (p=0.12), and that at 10 degrees 4.7±1.4/4.6±1.3 (p=0.10). No statistical difference was observed between the operative and contralateral side (Fig. **4**).

## DISCUSSION

One of the important points that this current study validates is the superior postoperative values of muscular strength with BHP use compared to CM nailing. Previous articles that have compared CM nailing and BHP procedures focused on the degree of surgical invasion, length of operative time, the need for blood transfusion, length of hospitalization, and mortality rates [6, 10, 11]. Although, a few articles included functional results, our study concentrated on postoperative muscle strength which no articles described, to our knowledge.

Evaluation of postoperative muscle strength has not been given much attention by most trauma surgeons. One reason may be that the major hip scoring system does not include postoperative muscle strength as an item in the list. To cite an example, the Harris Hip Score (HHS), widely used for hip fracture evaluation, assigns 44 points for pain, 47 points for function, 5 points for range of motion, and 4 points for deformity [14]. The functional score which partially reflects muscle strength, consists of a simple questionnaire on the patient’s daily activities [15, 16]. Muscle strength is not exclusively evaluated and is not given any score.

In our experience, patients who were in the younger age group, and those who were relatively healthier did not complain of any significant muscle weakness, even if their muscular strength was substantially weakened. This means active patients were able to adapt well to their postoperative states and developed sufficient compensatory strategies for using the surrounding hip muscles. However, the number of patients complaining of muscle weakness in the operated side was increasing, as elderly patients gradually noticed their impairment. Our impression coincides with Zlowodski M *et al.* [17] who cautioned patients to have a potential risk of future functional retardation. With this study, we hope that more orthopedic trauma surgeons would pay closer attention to examine muscle strength during the follow-up period.

The amount of decrease in muscular strength that may significantly affect postoperative functioning is not known as the current literature lacks this information. Patients with postoperative shortening of the abductor moment arm by at least 5mm among patients with femoral neck fractures have been reported to have significantly lower Short Form-36 (SF-36) scores [18]. From a rehabilitation standpoint, decreased lower extremity strength had a negative influence on maintaining upright balance during the stance phase of walking speed [19]. Shortening of the femoral neck was the only significant variable that was predictive of a low SF-36 physical functioning score [17]. Although the articles mentioned above are not enough to deduce the degree of muscular weakness causing functional deficit, they provide information on functional disorder following declined muscular strength. Following this preliminary work, further study will be aimed to find minimal or moderate functional deficits among patients with femoral intertrochanteric fractures.

The reduction in abduction strength after CM nailing may be attributed to 3 causes. First, although sliding towards the fracture site may be beneficial to fracture healing and accelerates the rehabilitation program, the negative effects of sliding or “shortening” are considered to contribute to losing muscular strength [3]. Since the gluteus medius is shorter than the surrounding iliopsoas, gluteus maximus, and quadriceps, the same amount of shortening caused more damage on the gluteus medius [17]. It has also been demonstrated that shortening of the femoral neck after osteosynthesis significantly decreases abduction strength, while flexion, extension, and adduction strengths were fairly maintained [17, 18, 20]. Second, reaming of the greater trochanter for gamma nail insertion may cause injury to the gluteus medius tendons. In a cadaveric study of 34 specimens, McConnell *et al.* [21] documented a 27% incidence of injury to the gluteus medius tendon insertion after using a 17 mm reamer, with the entry point of the gamma nail at the tip of the greater trochanter. On the contrary, Perez *et al.* [22] disputed no damage to the gluteus medius tendon in their cadaveric study using a modified medial trochanteric portal. Nevertheless, their entry point was located slightly medial to the tip of the greater trochanter and the 14 mm diameter of the reamer was smaller for conventional CM nailing. Therefore, their result cannot be applied for routine surgery with CM nailing. Meanwhile, it could be argued that nail insertion with minimal tissue exposure could be beneficial [3], although a small incision does not always guarantee the absence of damage to surrounding hip structures. Therefore, damage to the gluteus medius tendon reportedly seems unavoidable with gamma nail insertion procedures and injury to this muscle should be recognized as a potential cause of postoperative muscle weakness. Third, proximal migration of the greater trochanter may compromise abductor function, impairing the patient’s ability to walk normally [23, 24]. However, interpretation of a displaced greater trochanter in plain radiographs may be inaccurate due to overlapping radiographic views of the nail implant on the greater trochanter. Our study was unable to address this subject as well. Several factors are involved in causing postoperative muscular weakness.

BHP may offer several advantages over internal fixation such as lower rates of complications related to lag screw cutout and reoperation, as occasionally observed for unstable and comminuted intertrochanteric fracture fixed with CM nails [6, 9, 10]. However, one of the major drawbacks of ordinary BHP was greater trochanter non-union. Fichman *et al.* [9] proposed that a better technique for greater trochanter fixation, and not the conventional transfixing bolts, wires, or plates could lower the incidence of non-union, and added that active leg abduction restriction for eight weeks allows for healing of the abductor mechanism. In this current research, we proved that most of the abduction force could be maintained postoperatively with this novel BHP without any radiographic sign of non-union. With this product, orthopedic surgeons have lesser concerns regarding a displaced greater trochanter possibly causing a decrease in abduction force postoperatively.

There are several limitations to our current study. First, the sample size was relatively small. This is attributed to our stringent inclusion and exclusion criteria. However, the follow-up rate was approximately 90%, which is much higher than in other similar studies [20]. During the entire follow-up period, all patients were examined either at the hospital or at nursing homes where we visited them for measurements. Second, the minimal follow-up period was set to almost one year not extending to two years. Since not a few patients were excluded after two years due to aging and combined spinal or central nerve disorders, a longer follow-up period set to five years as longest would be inappropriate to reach a generalized conclusion in such an aged group. According to our other data of some patients, successive follow up study twice reveals result of continued decline of abduction strength. Third, although patients were age- and sex-matched, the BHP group was selected from a different database. Thus, the two therapists in charge of muscular measurement could not be blinded to the patients’ implants. However, the population characteristics were very similar, which prevented confounding variables that would have otherwise been present in a prospective randomization study. Fourth, the BHP procedure itself is difficult and requires experience and well-trained surgical skills [11]. Fifth, the two therapists in charge of muscular measurement were not blinded to the patients’ implant.

## CONCLUSION

Although there are several limitations in our preliminary study, CM nailing causes a 25-30% decrease in muscle strength around the hip joint, particularly during abduction. This decrease may significantly affect postoperative functioning. With this BHP, postoperative muscle strength during abduction is maintained due to better trochanter reduction and accelerated weight bearing. We understand that surgeons should consider all factors related to during and postoperative course including postoperative muscle weakness, in selecting the appropriate surgical procedure for femoral intertrochanteric fractures. However, this decline possibly continues for even more than a decade, as observed in some of our patients (not included in this case series). This difference in muscle strength can lead to imbalances in patients and may lead to falls that must be proved or given as hypothesis for a new study.

## Figures and Tables

**Table 1:** Patient Demographics of two groups, CM nailing and BHP.

	****CM nailing	BHP	P value
Age at the surgery* (yr)	84 (73~91)	85 (77~93)	0.19
Number of patients	20	20	1.0
Male (no.)	2	2	1.0
Postope. Followup*(days)	580(330~829)	555(326~1514)	0.38
Fracture type (AO/OTA)			
A-1	10	8	0.59
A-2	10	12	

*The values are given as the mean.
